# Red flags for a concomitant giant cell arteritis in patients with vertebrobasilar stroke: a cross-sectional study and systematic review

**DOI:** 10.1007/s13760-020-01344-z

**Published:** 2020-04-22

**Authors:** Ahmed Mohamed Elhfnawy, Doaa Elsalamawy, Mervat Abdelraouf, Mira Schliesser, Jens Volkmann, Felix Fluri

**Affiliations:** 1 Department of Neurology, University Hospital Würzburg, Josef-Schneider Street 11, 97080 Würzburg, Germany; 2 Department of Neurology, University Hospital of Alexandria, Alexandria, Egypt; 3 Department of Neurology, University Hospital of Essen, Essen, Germany

**Keywords:** Giant cell arteritis, Vertebrobasilar stroke, Blood sedimentation, C-reactive protein, Hemoglobin, Stenosis

## Abstract

Giant cell arteritis (GCA) may affect the brain-supplying arteries, resulting in ischemic stroke, whereby the vertebrobasilar territory is most often involved. Since etiology is unknown in 25% of stroke patients and GCA is hardly considered as a cause, we examined in a pilot study, whether screening for GCA after vertebrobasilar stroke might unmask an otherwise missed disease. Consecutive patients with vertebrobasilar stroke were prospectively screened for GCA using erythrocyte sedimentation rate (ESR), C-reactive protein (CRP), hemoglobin, and halo sign of the temporal and vertebral artery on ultrasound. Furthermore, we conducted a systematic literature review for relevant studies. Sixty-five patients were included, and two patients (3.1%) were diagnosed with GCA. Patients with GCA were older in age (median 85 versus 69 years, *p* = 0.02). ESR and CRP were significantly increased and hemoglobin was significantly lower in GCA patients compared to non-GCA patients (median, 75 versus 11 mm in 1 h, *p* = 0.001; 3.84 versus 0.25 mg/dl, *p* = 0.01, 10.4 versus 14.6 mg/dl, *p* = 0.003, respectively). Multiple stenoses/occlusions in the vertebrobasilar territory affected our two GCA patients (100%), but only five (7.9%) non-GCA patients (*p* = 0.01). Our literature review identified 13 articles with 136 stroke patients with concomitant GCA. Those were old in age. Headache, increased inflammatory markers, and anemia were frequently reported. Multiple stenoses/occlusions in the vertebrobasilar territory affected around 70% of stroke patients with GCA. Increased inflammatory markers, older age, anemia, and multiple stenoses/occlusions in the vertebrobasilar territory may be regarded as red flags for GCA among patients with vertebrobasilar stroke.

## Introduction

Giant cell arteritis (GCA) can be diagnosed if at least three out of the following American College of Rheumatology criteria are met: age >  = 50 years, new-onset localized headache, tenderness or reduced pulsation of the temporal artery (TA), increased erythrocyte sedimentation rate (ESR) >  = 50 mm in the first hour and/or positive TA biopsy [1]. These features may also occur in stroke patients but may be underestimated; stroke survivors are usually old in age, headache after stroke does often not receive much attention and increased inflammatory markers after an ischemic stroke are usually attributed to aspiration pneumonia or non-specific infection. Headache affects 7–28% of patients with stroke [2–4], and vertebrobasilar stroke (VB-stroke) is more frequently associated with headache [2–4]. Ischemic stroke affects 3–6% of patients with GCA [5, 6] and occurs in up to 73% of these patients in the VB-territory [7]. In at least one-fourth of patients with ischemic stroke, the etiology remains undetermined [8]. Moreover, the incidence of GCA among patients with stroke is unknown. Screening studies for GCA among patients with ischemic stroke are sparse. A halo sign or concentric thickening of the vascular wall on ultrasound examination of the TA is highly specific for GCA [9–11]. Previous studies found a sensitivity and specificity of the halo sign of 54–92% and 81–96%, respectively [9–11], in comparison to the respective values of the TA biopsy with 39–43% and 100% for GCA [9, 10]. Furthermore, in a previous meta-analysis, the specificity of ultrasound was found to increase to 100%, if bilateral halo signs are detected [11]. Of note, halo sign has been mainly investigated in relation to temporal artery involvement in classical cranial GCA but not in other subtypes of GCA [12]. Recently, the European League Against Rheumatism (EULAR) recommended ultrasound examination of the TA as a first-line imaging modality in patients with suspected predominantly cranial GCA [13]. With these considerations in mind, we investigated, whether screening for a halo sign of the TA and vertebral artery (VA) as well as for inflammatory markers in patients with VB-stroke is useful to unmask a concomitant GCA.

## Materials and methods

In a cross-sectional study, consecutive patients admitted to the Department of Neurology (University Hospital of Würzburg) with the diagnosis of VB-stroke were prospectively screened for the presence of halo sign of the extracranial VA and TA on both sides between February and October 2018. Additionally, routine laboratory investigations were performed, namely C-reactive protein (CRP), ESR, and hemoglobin. Ultrasound examination was conducted on a Toshiba AplioXG machine (Toshiba Medical Systems Corporation, Tochigi, Japan). Both VAs were examined in the semi-lying position with the neck slightly extended and turned to the other side using a 7.5-MHz linear transducer in at least three various levels for the presence of halo sign in the color-coded mode. Stenosis in the VB-territory was defined as the presence of a segmentally increased flow velocity, whereas an occlusion was defined as lack of blood flow or the detection of an occlusion signal on ultrasound examination. The TA was examined in the power mode using a 12-MHz linear transducer. First, the proximal part of the TA was identified according to its anatomical landmark directly in front of the ear and was then traced distally. Halo sign was defined according to the definition of the Outcome Measures in Rheumatology Clinical Trials (OMERACT) as a concentric well-delineated homogenous hypoechoic alteration of the arterial wall, visible in the longitudinal and transverse scans [14]. Atherosclerosis of the carotid artery was defined as the presence of a visible plaque of at least 2 mm thickness, protruding into the vascular lumen. Two non-blinded examiners (AME and MS) performed the ultrasound examinations.

### Systematic literature review

We conducted a literature search on 27/12/2019 with no restriction to publication date. We searched the database “Pubmed” for English-language sources using the following keywords: “Stroke” and “giant cell arteritis”.

We conducted two reviews of the literature; the first one was performed to search for red flags of GCA among patients with ischemic stroke and the second one was carried out to identify the prevalence of GCA among patients with stroke. Only primary sources were included. The following inclusion criteria were applied: (1) English language of the article, (2) diagnosis of ischemic stroke and GCA, (3) availability of clinical data at the time of ischemic stroke diagnosis, (4) availability of full text, and (5) publication of the article in a peer-reviewed journal. We excluded case reports, studies with less than three cases, review articles or any other articles lacking the clinical data of the patients at the time of stroke diagnosis.

We extracted the following data: Author name, publication year, journal name, age and sex of the patient, occurrence of the ischemic stroke in the VB-territory, headache, visual manifestations, ESR, CRP, hemoglobin, and detection of multiple stenosis/occlusions in the VB-territory.

### Statistics

Quantitative data were expressed using median and range, while qualitative data were expressed in absolute values and percentages. To check for normality, we used QQ-plot, histogram, and the Shapiro–Wilk test. We used Fisher’s exact test for categorical data and Mann–Whitney *U*-test for continuous data. Data were analyzed in SPSS software package version 25 (SPSS, Chicago, IL, USA). *P*-values < 0.05 were considered statistically significant.

## Results

Baseline data are shown in Table 1. Of the 65 screened patients, halo sign of both TAs and at least one VA was detected in the two patients (3.1%) who were diagnosed with GCA, whereas the remaining patients (*n* = 63) did not show halo sign of the examined arteries. The two cases with GCA are discussed below.

**Table 1:** Characteristics of the patients in current study

Characteristic	All (*n* = 65)	No GCA (*n* = 63)	GCA (*n* = 2)	*P*-value
Baseline				
Age, years	69 (31–90)	69 (31–88)	85 (80–90)	0.02*
Female sex	23 (35%)	22 (35%)	1 (50%)	0.59
Hypertension	54 (83.1%)	52 (82.5%)	2 (100%)	0.69
Diabetes mellitus	8 (12.3%)	7 (11.1%)	1 (50%)	0.23
Atrial fibrillation	20 (30.8%)	19 (30.2%)	1 (50%)	0.52
Active smoker	12 (18.5%)	12 (19%)	0 (0%)	0.66
Previous stroke	12 (18.5%)	12 (19%)	0 (0%)	0.66
Clinical				
Acute onset of any headache^a^	20 (30.8%) ^b^	20 (31.7%)	0 (0%)	0.46
Acute onset of temporal headache^a^	4 (6.2%)^b^	4 (6.3%)	0 (0%)	0.87
Temporal tenderness	3 (4.6%)^b^	3 (4.8%)	0 (0%)	0.91
Thickened temporal artery	6 (9.2%)	5 (7.9%)	1 (50%)	0.18
History of amaurosis fugax	2 (3.1%)	2 (3.2%)	0 (0%)	1
NIHSS on admission	2 (0–42)	2 (0–42)	3 (0–6)	0.48
Good outcome on discharge^c^	50 (76.9%)	49 (77.8%)	1 (50%)	0.41
Laboratory investigations				
ESR (mm after 1 h)	12 (1–100)	11 (1–60)	75 (50–100)	0.001*
CRP (mg/dl)	0.25 (0.02–7.09)	0.25 (0.02–7.09)	3.84 (3.07–4.61)	0.01*
Hemoglobin (g/dl)	14.5 (8.2–18.6)	14.6 (8.2–18.6)	10.4 (10–10.8)	0.003*
Platelets (n*1000/µl)	246 (85–1272)	242 (85–1272)	315 (291–338)	0.09
Stenosis and/or occlusion in the vertebrobasilar territory				
One	21 (32.3%)	19 (30.2%)	2 (100%)	0.1
> = 2	7 (10.8%)	5 (7.9%)	2 (100%)	0.01*
Atherosclerosis of the internal carotid artery with stenosis < 50%	42 (64.6%)	40 (63.5%)	2 (100%)	0.41
Atherosclerosis of the internal carotid artery with stenosis ≥ 50%	4 (6.2%)	3 (4.8%)	1 (50%)	0.12

Results are expressed in absolute values (percentage) or median (range)

*CRP* C-reactive protein, *ESR* erythrocyte sedimentation rate, *GCA *giant cell arteritis, *NIHSS* national institute of health stroke scale

*Statistically significant results

^a^Acute onset was defined as headache occurring 3 days before or after stroke

^b^In three non-GCA patients, history regarding headache was not available and assessment of the temporal tenderness was not possible because of the bad general condition

^c^Good outcome on discharge was defined as mRS ≤ 2

### Case 1

**Fig. 1:**
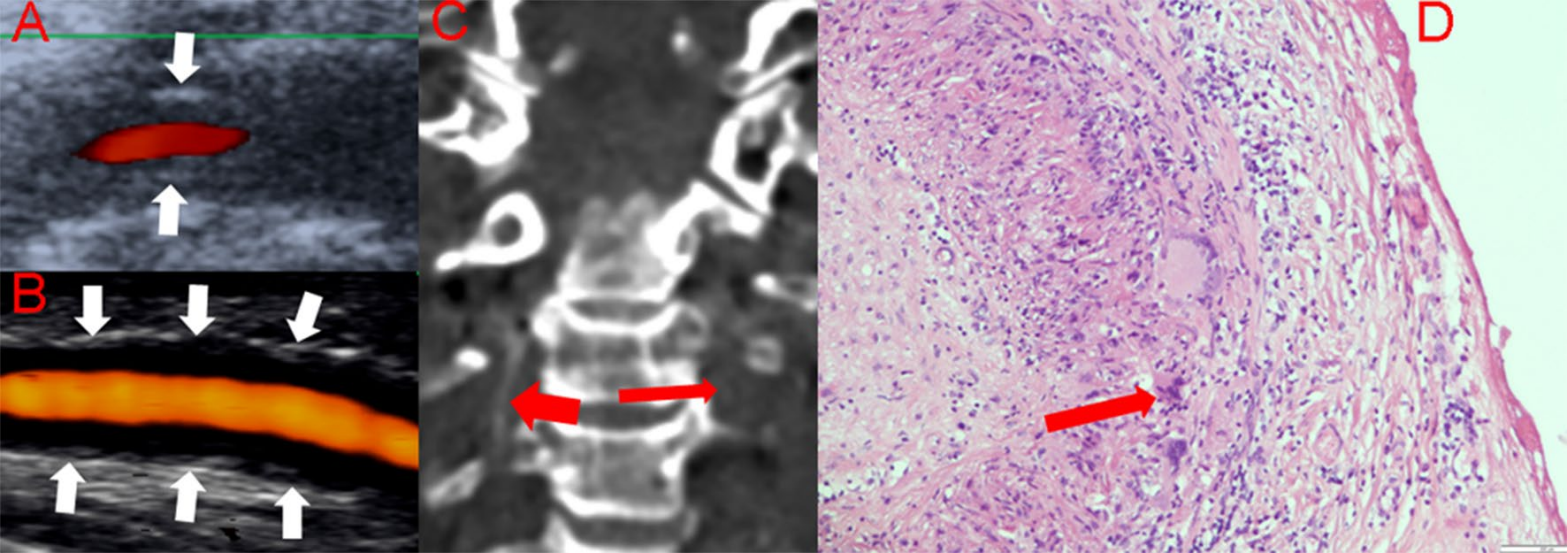
**a**, **b** and **c** refer to the first patient with giant cell arteries (GCA). **a** Color-coded duplex examination showing halo sign (hypoechogenicity of the vessel wall) in the left vertebral artery (white arrows). **b** Ultrasound examination in the power mode showing halo sign of the temporal artery on the right side (white arrows). **c** Computer tomographic angiogram showing stenosis in the right vertebral artery, representing the “string-of-beads” sign (short thick red arrow) and occlusion of the left vertebral artery (long thin red arrow). **d** Haematoxylin and eosin staining of a biopsy from the temporal artery of the second patient with GCA showing transmural infiltration of the all wall layers with mixed inflammatory cells consisting of lymphocytes and plasma cells with multinucleated giant cells (long red arrow) (color figure online)

A 90-year-old male patient presented with acute onset dysarthria and instability of gait. The patient has experienced a deterioration of his general state of health; the relatives observed a disturbed level of consciousness over the past 6 months. The patient was known to suffer from hypertension, dyslipidemia, and atrial fibrillation and was on edoxaban; three months before admission, the dose of edoxaban was reduced from 60 to 30 mg/d because the patient was frequently found to have a sinus rhythm in the electrocardiogram (ECG). Previous or new-onset headache was denied. On admission, the patient was disoriented, restless and dysarthric without evidence of other focal neurological signs. TAs were thickened but not tender to palpation. ECG on admission showed no atrial fibrillation. ESR was 50 mm after one hour, CRP was 3.07 mg/dl (reference value 0–0.5 mg/dl) and hemoglobin was 10.8 mg/dl (reference value 14–18 mg/dl). Cerebral computed tomography (CT) revealed an acute pontine and cerebellar infarction. On ultrasonography, an evident halo sign was seen around both VAs and TAs (Fig. 1a, b). The CT-angiography showed multiple stenotic segments of the right VA in all four segments, bringing to the mind the “string-of-beads” sign (Fig. 1c). Because of the patient’s old age and poor general state of health, we refrained from further diagnostic workup for ethical reasons and placed the patient on palliative treatment. The patient died 6 days after admission and received no immunosuppressive treatment for GCA.

### Case 2

An 80-year-old female patient presented with a transient acute onset of word-finding difficulty for 10 min. Four and two weeks before admission, she developed nausea and vomiting, which necessitated hospital admission. Previous or new-onset headache was denied. Clinical examination revealed a normal finding. TAs were neither thickened nor tender to palpation. The patient suffered from diabetes mellitus and hypertension. ESR was 100 mm after one hour, CRP was 4.61 mg/dl and hemoglobin was 9.8 g/dl. The magnetic resonance imaging showed subacute right cerebellar infarction. Ultrasonography revealed focal stenosis (maximum flow velocity 142 cm/s) and halo sign in the vertebral segment of the right VA, occlusion of the left VA and halo sign around both TAs. TA biopsy showed transmural infiltration of all vascular wall layers with mixed inflammatory cells consisting of lymphocytes and plasma cells with multinucleated giant cells (Fig. 1d). Prednisolone 60 mg/d and methotrexate 15 mg/d were started. On discharge, we recommended the slow gradual withdrawal of prednisolone under strict monitoring of CRP and ESR in the family physician’s office. Furthermore, the patient was placed on antiplatelet treatment with acetylsalicylic acid 100 mg/d. Two months later, in a follow-up visit in our neurovascular clinic, the patient was on prednisolone 15 mg/d and methotrexate 15 mg/d and the inflammatory markers were markedly increased (CRP 9 mg/dl and ESR 85 mm after one hour). We increased the dose of prednisolone to 20 mg/d. Two weeks later, the patient developed atypical pneumonia with bilateral infiltrates and ultimately died. The trade-off between the risk of relapse under lower steroid doses and the immunosuppressive side effects of higher steroid doses is a matter of discussion.

### Results of the literature research

Our reported search strategy identified 308 publications that were assessed for our inclusion criteria. We conducted two different literature reviews. The first review encompassed 13 articles (136 patients) which reported the criteria of patients with stroke and concomitant GCA at the times of stroke diagnosis. We identified possible red flags as shown in Table 2. Overall, patients were old in age with similar sex distribution. In more than two-third of patients with stroke and concomitant GCA, the VB-territory was affected. Headache and/or facial pain were reported in more than two-third of the cases. Most patients had increased inflammatory markers (CRP and/or ESR) and suffered from anemia. About 70% of patients had multiple stenoses/occlusions in the VB-territory.

**Table 2:** Characteristics of patients with giant cell arteritis at the times of stroke diagnosis in the included studies

Author, journal	*N*	Age (years)	Male sex, *n* (%)	Affection of the VB- territory, *n* (%)	Headache, *n* (%)	ESR (mm after 1 h) or *n* (%)	CRP (mg/dl) or *n* (%)	Hemoglobin (g/dl)	Multiple stenoses/occlusions in the VB- territory, *n* (%)
Pariente et al. [16], J Autoimmun	18	Median (range) 83 (67–96)	11 (61.1%)	11 (61.1%)	15 (83.3%)	NA	Median (range) 6.6 (1–21.1), increased in 15 (83.3%)	NA	NA, but bilateral in 6 (33.3%)
Conway et al. [32], Stroke	14	70 (63–78)	7 (50%)	NA	8 (57.1%)	53 (36–88), increased in 11 (78.6%)	4.8 (1.8–10.9), increased in 12 (85.7%)	NA	NA
Lago et al. [33], Neurologia	6	71 (63–73)	3 (50%)	4 (66.7%)	4 (66.7%)	68 (16.5–88.5), increased in 5 (83.3%)	4.9 (1.7–6.4) Increased in 6 (100%)	Anemia in 2 patients, for the other 4 patients NA	3 (50%)
Chazal et al. [20], Joint Bone Spine	14 (1 with TIA)	Median (range) 73.5 (65–82)	3 (21.4%)	6 (42.9%)	5 (35.7%)	NA	Median (range) 0.6 (0.2–6.8)	NA	NA
de Boysson et al. [7], J Rheumatol	40	Median (range) 78 (60–91)	19 (47.5%)	29 (72.5%)	29 (72.5%)	Median (range) 68 (10–119)	Median (range) 6.1 (2.8–18.5)	Anemia in 22/37 (59.5%), in 3 patients data na	9/12 (75%), in the other patients data NA
Alsolaimani et al. [26], J Rheumatol	5	70 (53–78)	3 (60%)	3 (60%)	5 (100%)	37 (27.5–91), increased in 3 (60%)	2.1 (0.9–3.5) increased in 3 (75%), in 1 patient na	NA	4 (80%)
Larivière et al. [21], Medicine (Baltimore)	8	72 (62–76)	6 (75%)	7 (87.5%)	6 (75%)	NA	7.3 (4.6–10.7)	NA	4 (50%)
Samson et al. [34], J Neurol Neurosurg Psychiatry	4	82 (80–88)	3 (75%)	3 (75%)	1 (25%)	56 (42.5–66.5), increased in 2 (50%)	4.7 (3.3–19.6), increased in 4 (100%)	NA	NA
Zenon et al. [6], Rheumatol Int	6	77 (64–83)	2 (33.3%)	2 (33.3%)	5 (83.3%)	93 (72–100), increased in 6 (100%)	9.4 (5.3–12.8), increased in 6 (100%)	NA	NA
García-García et al. [35], Stroke	5	79 (77–82)	2 (40%)	5 (100%)	2 (40%)	60 (40.5–92.5), increased in 4 (80%)	NA	NA	2 (40%)
Boettinger et al. [36], BMJ Case Rep	3	73, 74, 65	2 (66.7%)	3 (100%)	2 (66.7%)	85, 100, in one patient na	2.6, 2.3, in one patient NA	NA	3 (100%)
Solans-Laqué et al. [17], Medicine (Baltimore)	7	74 (67–85)	4 (57.1%)	5 (71.4%)	5 (71.4%)	98 (78–99)	NA	10.5 (9–11), anemia in 7 (100%) patients	2/6 (33.3%) (in one patient data NA)
Wiszniewska et al. [25], Cerebrovasc Dis	6	80 (74–81)	5 (83.3%)	4 (66.7%)	6 (100%)	37.5 (9.5–82.5), increased in 3 (50%)	NA	NA	NA

All results are expressed as median (interquartile range) or absolute numbers (%), unless otherwise specified

*CRP* C-reactive protein, *ESR* erythrocyte sedimentation rate, *NA *not available, *TIA* transient ischemic attack, *VB* vertebrobasilar

CRP was considered as increased, if the value was > 0.5 mg/dl

ESR was considered increased if the value was > age/2 for men or age/2 + 10 for women

In the second literature review, two articles (5359 patients) reporting the prevalence of GCA among patients with stroke were identified (Table 3). In one study, 1273 patients with stroke, either in the anterior or VB-territory, were screened for halo sign of the VA. Five patients with ischemic stroke (all in the VB-territory) were diagnosed with GCA. No data were available, whether the ischemic stroke was located in the anterior or VB-territory among the screened patients. Since VB-stroke represents 15–20% of all ischemic strokes [7], the prevalence of GCA might be estimated at 2–2.6% among patients with VB-stroke in the aforementioned cohort. In another study, 4086 patients with either hemorrhagic or ischemic stroke were recruited. The patients were not actively screened for GCA. Six patients with ischemic stroke (four in the VB-territory) were diagnosed with GCA.

**Table 3:** Studies reporting the prevalence of GCA among patients with stroke

Author, journal	Total no. of patients with stroke	Type of stroke	Screening method	Study period	Cases diagnosed with GCA	Comment
García-García et al. [35], Stroke	1273 patients	Ischemic stroke, either in the anterior or VB-territory	Halo sign of the vertebral artery	Between Mar 2008 to Jan 2010	5 patients (0.4%); all in the VB-territory	No data are available regarding the stroke location (anterior or VB-territory) in the screened patients. Assuming that VB-stroke represents 15–20% of all ischemic strokes, the prevalence of GCA might be estimated as 2–2.6% among patients with VB-stroke
Wiszniewska et al. [25], Cerebrovasc Dis	4086 patients in the Lausanne Stroke Registry	First stroke, either ischemic or hemorrhagic	No screening applied, routine work-up	between Jan 1980, and Dec 1998	6 patients (0.15%) with ischemic stroke, four of them with affection of the VB-territory	No data are available regarding the stroke type (ischemic or hemorrhagic) or location (anterior or VB-territory) among the screened patients

*GCA* giant cell arteritis, *VB* vertebrobasilar

## Discussion

In this pilot study, GCA was diagnosed among 3.1% of patients with VB-stroke. VB-stroke patients with GCA were older in age, had increased ESR- and CRP-values with decreased hemoglobin-values, and were more likely to have >  = 2 vertebrobasilar stenoses/occlusions. This study is the first one to provide red flags for GCA among patients with VB-stroke. Moreover, the current cohort is one of the scarce available prospectively collected GCA-cohorts.

Our patients with GCA were significantly older than those without GCA. The median age of the two patients diagnosed with GCA was 85 years. Similarly, a median age of 78–83 years for stroke patients with concomitant GCA was previously reported [7, 15, 16]. In our literature review, the patients were usually older in age (Table 2).

In our literature review, we found that headache was reported among nearly two-thirds of patients with stroke and concomitant GCA (Table 2). Our two patients with GCA did not have headache. Among our non-GCA patients, acute onset headache within 3 days before or after stroke was found in 20/63 (31.7%) of the patients. Previous studies found headache in association with acute stroke among 7–28% of the patients [2–4]. Younger patients, as well as patients with stroke in the VB-territory, are more likely to have headache at stroke onset [2–4]. Of note, headache at stroke onset was found to predict good outcome after stroke [4].

Around 70% of the reported cases with stroke and concomitant GCA had multiple stenoses/occlusions in the VB-territory (Table 2). Of note, the use of different examination modalities, i.e. neurovascular ultrasound, CTA, MRA, and/or DSA as well as the retrospective nature might explain the low incidence of multiple stenoses/occlusions in the VB-territory (33.3%) reported in some studies [16, 17]. A previous retrospective multicenter study reported multiple stenoses in the VB-territory among 9/12 (75%) of patients with GCA-related VB-stroke [7]. In line with the authors of the aforementioned study, we also speculate that the vascular stenoses/occlusions in our two reported cases with GCA may reflect an inflammatory process in the vascular wall rather than atherosclerosis. On the other hand, both patients with GCA had atherosclerotic plaques in the carotid artery, which is known to affect around 30% of the normal middle-aged population [18], and might indicate that the aforementioned wall alterations of the VAs are nevertheless of atherosclerotic origin. Furthermore, one might postulate that the concomitant occurrence of atherosclerosis and vasculitis significantly increases the risk of stroke. Nevertheless, a previous study on 40 patients with biopsy-proven GCA, who receiving steroid therapy, demonstrated that intima-media-thickness is not increased in GCA patients in comparison to matched controls [19]. Therefore, the susceptibility to develop a stroke in the vertebrobasilar territory in GCA patients may be the result of complex interaction between traditional cardiovascular risk factors and the disease itself.

The occurrence of stroke in GCA patients is usually associated with a poor prognosis; the mortality rate in these patients is 14–28% [7, 15, 16, 20]. In contrast, another study reported a remission rate as high as 75% among those patients [21]. In the current cohort, the first GCA patient died during hospital admission and the second one died two months later. Among the other 63 patients, one patient died during hospital admission and the other one died around two months after discharge. Of note, stroke recurrence among patients with GCA, despite immunotherapy, is reported [22, 23].

We diagnosed GCA in 3.1% of our patients with VB-stroke. In a Spanish cohort, GCA was diagnosed among 5/1237 (0.4%) patients with ischemic stroke [15]. This Spanish cohort was conducted on patients with stroke, either in the anterior or posterior circulation. Interestingly, the stroke of the five patients with concomitant GCA occurred in the VB-territory. The authors of the aforementioned study screened their patients using ultrasonography of the VA. We additionally examined the TA. Of note, the current study was carried out in Germany; the incidence of GCA in Northern Europe is higher than in Southern Europe [24]. In another cohort of 4086 with either hemorrhagic or ischemic stroke, six patients with ischemic stroke (four in the VB-territory) showed a concomitant GCA [25]. These patients were not actively screened for GCA; the diagnosis of GCA was rather established during the routine work-up. On the other hand, vertebrobasilar ischemia in GCA may be more common than what is reported clinically. In this regard, biopsy-proven GCA is usually associated with audiovestibular impairment, which may occur as a result of vasculitic-related ischemia involving the vertebrobasilar territory with affection of the terminal cochleovestibular vessels [27]. High blood supply is mandatory for optimal auditory and vestibular functions. The inner ear is supplied from the labyrinthine artery, which originates from the anterior inferior cerebellar artery [27]. These data further support the vasculitic involvement of the vertebrobasilar territory in patients with GCA. Furthermore, it might be speculated that GCA is underestimated among those patients due to the rare occurrence of typical cranial features of GCA like headache or visual disturbance. Similarly, previous studies showed that an “occult” GCA, manifesting as an isolated polymyalgia rheumatica or fever of unknown origin is not exceptional [28, 29]. In the current study, we screened our patients for the presence of halo sign. The latter was mostly studied in relation to temporal artery involvement in classical cranial GCA presenting with headache and visual disturbance [12]. Both of those symptoms were not present in our patients with GCA. Whether better screening methods might unmask more cases with “occult” GCA is a matter of future research.

Interestingly, a previous study investigated red flags for stroke among patients with GCA [30]. The authors identified 8 stroke patients (7 VB-strokes and one carotid) among 287 patients with biopsy-proven GCA. Among patients with GCA, irreversible visual loss and hypertension were predictors of stroke (both in the carotid and VB-territory). Conversely, female sex and anemia were protective against stroke (both in the carotid and VB-territory). Furthermore, smoking history predicted VB-stroke, whereas headache at the time of GCA diagnosis protected against VB-stroke. These results should be distinguished from ours because the authors aimed to identify high-risk criteria predicting the occurrence of stroke among patients with GCA, whereas we aimed to detect high-risk criteria for a concomitant GCA among patients with VB-stroke.

**Fig. 2:**
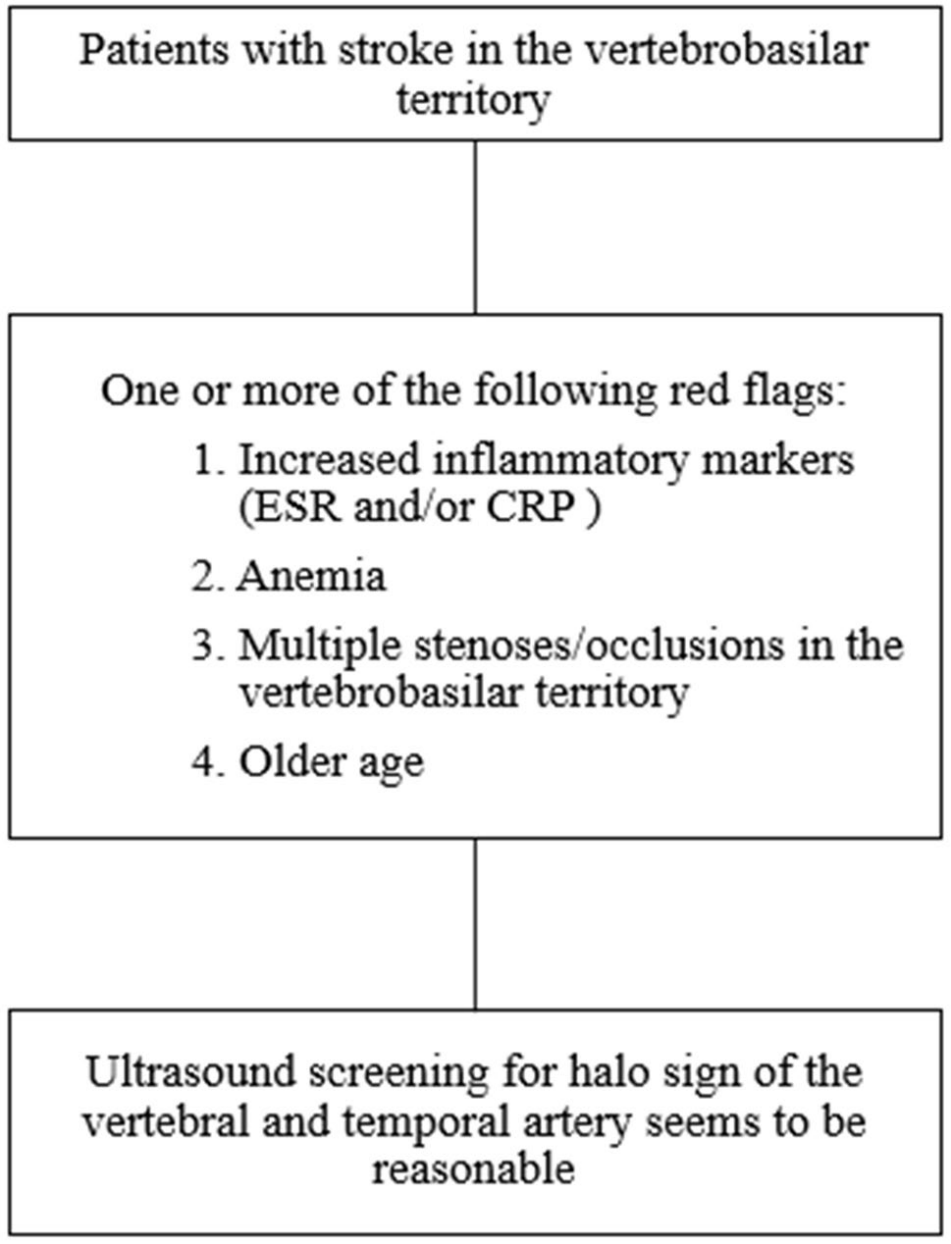
Proposed flow chart showing the red flags raising suspicion and warranting screeing for giant cell arteritis among patients with vertebrobasilar stroke

Most of the available research regarding GCA was conducted using a retrospective study design because GCA is a rare disease. The current study represents one of the very few available prospective GCA studies. Because of our prospective study design, we had only two available cases with GCA, which is the main limitation of the current work and makes it difficult to draw clinically significant conclusions. However, the findings of the current study along with the results of the literature review may serve as a proof-of-concept. Large epidemiological studies are needed to provide a proof-of-evidence in this regard. The occurrence of stroke in GCA patients is associated with a poor prognosis [7, 15, 16, 20]. Furthermore, GCA may result in visual loss, if not adequately treated and the mainstay treatment relies on immunomodulatory therapy [31]. Therefore, it seems to be reasonable to use non-invasive methods to screen for GCA among patients with VB-stroke, especially in the presence of increased inflammatory markers, anemia and/or multiple vascular stenoses/occlusions in the VB-territory (as shown in Fig. 2). In the presence of any of these parameters, a screening for halo sign of the VA and TA might be considered.

## Conclusion

The results of our literature review, as well as the findings of the current study, have shown that older age, increased inflammatory markers, anemia and/or the presence of multiple vascular stenoses/occlusions in the vertebrobasilar territory may be considered as red flags for GCA among patients with VB-stroke. A simple ultrasound examination for vertebral and temporal artery seems to be reasonable in VB-stroke with the aforementioned red flags.
