# Correction to Red flags for a concomitant giant cell arteritis in patients with vertebrobasilar stroke: a cross‑sectional study and systematic review

**DOI:** 10.1007/s13760-021-01763-6

**Published:** 2021-09-07

**Authors:** Ahmed Mohamed Elhfnawy, Doaa Elsalamawy, Mervat Abdelraouf, Mira Schliesser, Jens Volkmann, Felix Fluri

**Affiliations:** 1 Department of Neurology, University Hospital Würzburg, Josef-Schneider Street 11, 97080 Würzburg, Germany; 2 Department of Neurology, University Hospital of Alexandria, Alexandria, Egypt; 3 Department of Neurology, University Hospital of Essen, Essen, Germany

## Correction to: Acta Neurologica Belgica (2020) 120:1389–1398 10.1007/s13760-020-01344-z

The article “Red flags for a concomitant giant cell arteritis in patients with vertebrobasilar stroke: a cross‑sectional study and systematic review”, written by Ahmed Mohamed Elhfnawy, Doaa Elsalamawy, Mervat Abdelraouf, Mervat Abdelraouf, Jens Volkmann, and Felix Fluri, was originally published Online First without Open Access. After publication in volume 120, issue 6, page 1389–1398 the author decided to opt for Open Choice and to make the article an Open Access publication. Therefore, the copyright of the article has been changed to © The Author(s) 2021 and the article is forthwith distributed under the terms of the Creative Commons Attribution 4.0 International License, which permits use, sharing, adaptation, distribution and reproduction in any medium or format, as long as you give appropriate credit to the original author(s) and the source, provide a link to the Creative Commons licence, and indicate if changes were made. The images or other third party material in this article are included in the article’s Creative Commons licence, unless indicated otherwise in a credit line to the material. If material is not included in the article’s Creative Commons licence and your intended use is not permitted by statutory regulation or exceeds the permitted use, you will need to obtain permission directly from the copyright holder. To view a copy of this licence, visit http://creativecommons.org/licenses/by/4.0. Open access funding enabled and organized by Projekt DEAL.

The original article has been corrected.

